# Evaluating the Impact of Positive Fluid Balance on Mortality and Length of Stay in Septic Shock Patients

**DOI:** 10.7759/cureus.24809

**Published:** 2022-05-07

**Authors:** Nusrat Kharadi, Tooba Mehreen, Maria Habib, Ghulam Rasheed, Anum Ilyas, Aftab Akhtar, Kiran Abbas

**Affiliations:** 1 Department of Critical Care Medicine, Shifa International Hospital Islamabad, Islamabad, PAK; 2 Department of Internal Medicine, Shifa International Hospital Islamabad, Islamabad, PAK; 3 Department of Internal Medicine, Jinnah Postgraduate Medical Centre, Karachi, PAK

**Keywords:** sofa, severe sepsis, intensive care unit stay, emergency critical care, apache

## Abstract

Background

Sepsis is accountable for major morbidity and mortality in patients with critical illnesses. The present research was undertaken to evaluate the correlation between fluid balance during hospitalizations and patient outcomes in patients with sepsis.

Methods

An observational study was undertaken at the Critical Care Department, Shifa International Hospital, Islamabad, Pakistan between December 2021 to April 2022. The patients included were over the age of 17 years, with a proven diagnosis of infection. These patients either had positive bacterial cultures, suffered from organ failure secondary to sepsis, or had clinically evident signs of infection. Patients who were discharged during the period of study were eliminated from the study population. All patients were informed of the process and signed consent was obtained. Basic demographic information was recorded, which included the existence of any comorbid conditions, organ failure, medication use, or infection history. The severity of critical illness was determined for every single patient along with organ damage. The final patient outcome was recorded as in-hospital mortality.

Results

A total of 307 patients were included in the study with a total of 165 (53.75%) male patients. The overall mortality rate was 39.74%. The mean length of hospitalization was 17.42 ± 8.3 days. A high SOFA score was significantly associated with quartile 4 with a mean score of 14.1 (p < 0.001). Similarly, a significantly higher APACHE score was found in patients in quartile 4 (p < 0.001) thus indicating a relationship between severity of illness and positive fluid balance. Upon further assessment, it was found that the 28th day and 90th day were significantly greater in quartile 4 in comparison to other quartiles. Similarly, the overall length of stays in the hospital and in the ICU were also significantly associated with greater fluid balance (p < 0.001).

Conclusion

In our study, it was concluded that monitoring fluid balance in critically ill patients is very important. The highest 28-day and 90-day mortality were seen in patients with the greatest positive fluid balance. However, the cause of high mortality in this cohort could be multifactorial; therefore, the relationship of positive fluid balance with patient outcome remains debatable.

## Introduction

Sepsis is now regarded as a dysregulated inflammatory reaction and is accountable for substantial morbidity and mortality in patients with critical illness [1,2]. Sepsis leads to a significant reduction of blood volume, which results from inadequate fluid intake and uncompensated external losses, and leakage into the interstitial body spaces. Hence, large amounts of intravenous fluid are often required for cardiac output to rise and to facilitate enhanced peripheral blood flow [3]. However, guiding fluid therapy continues to be a challenging problem, since cardiac filling pressures are erratic and fluid response indications are not always clear to comprehend, and surveilling approaches are all restricted [4,5].

Many studies that have been carried out have demonstrated a link between positive fluid balance and mortality, however, the matter stands unresolved if this is merely a correlation or a cause and effect [5,6]. Acheampong and Vincent revealed that the average fluid intake per day was higher in individuals who died as compared to those who survived (p = 0.03). Furthermore, the authors noticed that the output volumes were similar in both groups, thus the end result was that the daily fluid balance was two times higher in expired patients than in survivors (p < 0.001) [7]. The FINNAKI study revealed that excessive fluid administration was correlated with a much higher 90-day mortality rate [8].

While there is increased recognition of the therapeutic importance of fluid overload, there is no unanimity regarding the effects of positive fluid balance in patients with critical illness with sepsis on their overall survival rates. Therefore, the present research was undertaken to evaluate the correlation between fluid balance during hospitalization and patient outcome in patients with sepsis.

## Materials and methods

A prospective observational study was undertaken at the Critical Care Department, Shifa International Hospital, Islamabad, Pakistan between December 2021 to April 2022. Ethical approval from the Institutional Review Board (IRB) Committee of Shifa International Hospitals Ltd. was procured with the reference # 340-21.

A non-probability convenience technique was employed to recruit participants in the study. By using the World Health Organization (WHO) sample size calculator, taking the frequency of mortality (42%) [9], in patients with a fluid balance of 0-5999 mL in 24 hours while taking the frequency of mortality (55%) in patients with a fluid balance of 18000-24000 mL in 24 hours, keeping confidence level (C.I) = 95%, margin of error = 8%, then the estimated sample size was n = 295.

The patients included in the research were over the age of 17 years, with a proven diagnosis of infection. These patients either had positive bacterial cultures, suffered from organ failure secondary to sepsis, or had clinically evident signs of infection as defined by a Sequential Organ Failure Assessment (SOFA) score of three or four. Patients who were discharged during the period of study were eliminated from the study population. The majority of the patients had gram-negative bacterial infections including Pseudomonas or E. coli, as well as gram-positive bacteria including staphylococci, and streptococci infections. A minority of the patients had fungal infections.

As per the International Sepsis Forum, sepsis was defined as damage to the body tissues and organs due to its own response to an infection [1]. All patients were informed of the process and signed consent was obtained. Basic demographic data were acquired from the participants, which included the existence of any comorbid conditions, organ failure, medication use, or infection history.

Acute Physiologic and Chronic Health Evaluation II (APACHE II) and SOFA scores were determined for every single patient. Any preexisting organ damage was noted. The APACHE II scoring system identifies the severity of the disease and is most frequently utilized in the ICU settings. On the other hand, the SOFA score is used to assess the patient’s progress and status during their stay in the ICU to accurately evaluate the development of organ dysfunction and subsequent failure. The final patient outcome was recorded as in-hospital mortality.

All recorded data were recorded and studied using Microsoft Excel and SPSS Statistics (IBM Corp., Armonk, NJ). All quantitative variables were presented as mean and standard deviation including patient’s age, fluid balance, mean input, and mean output. while all quantitative parameters were presented as frequency and percentages. The correlation between mortality and positive fluid balance among patients was determined using chi-square and one-way ANOVA where appropriate. A p-value of < 0.05 was considered the cut-off for statistical significance.

Fluid balance was determined by maintaining input and output charting. Fluid balance equates to the difference between output and input. Patients were subdivided according to the positive fluid balance into four quartiles as illustrated in Table 1. All data were recorded in a predefined proforma.

**Table 1:** Division of patients with fluid overload

Groups	Quartile 1	Quartile 2	Quartile 3	Quartile 4
Fluid balance at 24 hours	0-6199 mL	6200-12399 mL	12400-18,599 mL	18600-32000 mL
Frequency n (%)	177 (57.65%)	101 (32.90%)	19 (6.19%)	10 (3.26%)

## Results

This study consisted of a total of 307 individuals, with 165 (53.75%) male patients. The overall mortality rate was 39.74%. The mean length of hospitalization was 17.42 ± 8.3 days (Table 2).

**Table 2:** Characteristics of study participants

Patient Characteristics	Mean ± SD
Age in years	63.8 ± 7.5
Male gender, n (%)	165 (53.75%)
Body mass index in kg/m^2^	28.1 ± 9.1
Organ failure	
Hematologic, n (%)	66 (21.50%)
Respiratory, n (%)	148 (48.21%)
Metabolic / lactic acidosis, n (%)	100 (32.57%)
Kidney, n (%)	74 (24.10%)
Outcomes	
Hospital mortality	122 (39.74%)
Length of stay (LOS) at the hospital in days	17.42 ± 8.3
Comorbidities	
Chronic obstructive pulmonary disease (COPD), n (%)	38 (12.38%)
Diabetes, n (%)	52 (16.94%)
Immunocompromised, n (%)	28 (9.12%)

A high SOFA score was significantly associated with quartile 4 with a mean score of 14.1 (p < 0.001). Similarly, a significantly higher APACHE score was found in patients in quartile 4 (p < 0.001) thus indicating a relationship between severity of illness and positive fluid balance (Table 3).

**Table 3:** Association of positive fluid balance with sequential organ failure assessment (SOFA) and acute physiologic and chronic health evaluation II (APACHE II) scores

Group	SOFA Score (Mean ± SD)	p-value	APACHE II Score (Mean ± SD)	p-value
Quartile 1 (n=177)	9.7 ± 2.91	< 0.001	21.5 ± 7.1	< 0.001
Quartile 2 (n=101)	10.6 ± 2.72		24.9 ± 8.1	
Quartile 3 (n=19)	11.2 ± 3.9		27.8 ± 8.7	
Quartile 4 (n=10)	14.1 ± 3.7		28.3 ± 8.4	

Upon further assessment, it was found that the mortality rates on the 28th day and 90th day were significantly greater in quartile 4 in comparison to other quartiles. Similarly, the overall length of hospitalization in the intensive care unit was also significantly associated with greater fluid balance (p < 0.001) (Table 4). 

**Table 4:** Correlation of positive fluid balance with mortality rates and length of hospitalization and stay in the intensive care unit

	Quartile 1 (n=177)	Quartile 2 (n=101)	Quartile 3 (n=19)	Quartile 4 (n=10)	p-value
Mortality–n (%)					
28-day mortality	30 (16.95%)	29 (28.71%)	7 (36.84%)	4 (40.00%)	0.013
90-day mortality	64 (36.16%)	42 (41.58%)	10 (52.63%)	6 (60.00%)	0.176
Length of stay–days (mean ± SD)					
ICU length of stay	4.91 ± 2.02	5.58 ± 2.65	8.52 ± 3.76	11.0 ± 5.31	< 0.001
Hospital length of stay	16.7 ± 5.9	14.92 ± 6.89	20.67 ± 13.12	29.0 ± 15.3	< 0.001

## Discussion

Fluid resuscitation is an important element of sepsis management. Though positive fluid balance is essential, it has also been associated with poor prognosis and patient outcomes [7-10]. In our study, it was revealed that strong associations exist between mortality and positive fluid balance. Our results showed that a higher amount of fluid balance was significantly correlated with in-hospital deaths of patients suffering from sepsis. Additionally, the length of hospital stays and ICU admissions were also highest in patients who received the most fluids. However, it must be taken into consideration that higher APACHE and SOFA scores also relate to higher mortality, so we cannot attribute higher death rates in our patients to higher fluid volume alone [11].

In a similar study by Brotfain et al., it was found that fluid balance was a significant predictor of ICU deaths, and has a strong affiliation with multi-organ dysfunction following discharge in patients of septic shock [12]. Another study found that critically ill patients with a positive fluid balance had a much higher 28- and 90-day mortality, which is a finding consistent with our study. The study also revealed that positive fluid balance resulted in a greater dependency on mechanical ventilation [13]. This implies that fluid balance needs to be cautiously monitored in critically ill patients, as much higher volume overload has been found to decrease lung compliance thereby requiring additional respiratory effort [14].

There also exists a strong correlation between increased mortality and positive fluid balance in critically ill patients with concomitant renal failure. A study by Vaara et al. concluded that a fluid overload resulted in double the mortality [8]. Another study by Zoccali et al. assessed the correlation between fluid overload and mortality in 39,566 individuals with end-stage renal disease. The authors revealed that persistent fluid overload over a period of one year in individuals with end-stage renal disease is strongly related to an augmented risk for death [15].

Though positive fluid balance often corresponds with a worse prognosis, literature has also highlighted that a higher fluid resuscitation in the initial phase of the disease i.e. the first few days of ICU admission was associated with higher rates of survival and reducing the risks of ICU readmission following discharge [12]. However, no such finding was reported in our study. A similar finding was also noted in the study by Barmparas et al., which revealed that a positive fluid balance on the first day of ICU admission was correlated with reduced mortality rates [16]. A similar study by Shen et al. noted that a positive fluid balance on the following day, and not the first several hours of admission to ICU, was linked to higher in-hospital mortality. Thus, the study inferred that a positive fluid balance in the first several hours of ICU admission was associated with positive outcomes [17]. This implies that sufficient fluid administration in the initial phase of ICU administration followed by conservative fluid administration later may result in improved patient outcomes.

Contradictory findings were reported in a study by Cronhjort et al., which noted that no association was present between fluid balance and 90-day mortality. The lack of correlation indicates the possibility that fluid overload is only harmful beyond a set limit [18]. Our study was limited by a small sample size, which did not allow us to generalize our findings to a larger population. Further multicenter studies are required to explore the matter in more depth.

## Conclusions

Our study findings highlighted the significance of monitoring fluid balance in patients with critical illness and septic shock. However, whether positive fluid balance correlated with poor clinical outcomes in critically ill patients is still debatable. To validate the findings of the current study, it is imperative to explore the relationship of positive fluid balance with patient outcomes before labeling it as an effective means of evaluating the progress of patients admitted to the intensive care unit.
